# The Effect of a Standardized Ginger Extract on Chemotherapy-Induced Nausea-Related Quality of Life in Patients Undergoing Moderately or Highly Emetogenic Chemotherapy: A Double Blind, Randomized, Placebo Controlled Trial

**DOI:** 10.3390/nu9080867

**Published:** 2017-08-12

**Authors:** Wolfgang Marx, Alexandra L. McCarthy, Karin Ried, Dan McKavanagh, Luis Vitetta, Avni Sali, Anna Lohning, Elisabeth Isenring

**Affiliations:** 1 Faculty of Health Sciences and Medicine, Bond University, Gold Coast, QLD 4226, Australia; alohning@bond.edu.au (A.L.); lisenrin@bond.edu.au (E.I.); 2 Department of Nutrition and Dietetics, Princess Alexandra Hospital, Brisbane, QLD 4102, Australia; 3 National Institute of Integrative Medicine, Melbourne, VIC 3122, Australia; karinried@niim.com.au (K.R.); asali@niim.com.au (A.S.); 4 School of Allied Health, La Trobe University, Melbourne, VIC 3086, Australia; 5 Division of Cancer Services, Princess Alexandra Hospital, and Institute of Health and Biomedical Innovation, Brisbane, QLD 4102, Australia; alexandra.mccarthy@auckland.ac.nz; 6 School of Nursing, University of Auckland, Auckland 1010, New Zealand; Daniel.Mckavanagh@health.qld.gov.au; 7 School of Pharmacy, The University of Queensland, Brisbane, QLD 4072, Australia; 8 Sydney Medical School, The University of Sydney, Sydney, NSW 2006, Australia; luis.vitetta@sydney.edu.au; 9 Medlab Clinical Ltd., Alexandria, Sydney, NSW 2015, Australia

**Keywords:** ginger, nausea, CINV, dietary supplements, cancer, emesis

## Abstract

Ginger supplementation could be an effective adjuvant treatment for chemotherapy-induced nausea (CIN). The aim of this clinical trial was to address significant methodological limitations in previous trials. Patients (N = 51) were randomly allocated to receive either 1.2 g of standardised ginger extract or placebo per day, in addition to standard anti-emetic therapy, during the first three cycles of chemotherapy. The primary outcome was CIN-related quality of life (QoL) measured with the Functional Living Index- Emesis (FLIE) questionnaire. Secondary outcomes included acute and delayed nausea, vomiting, and retching as well as cancer-related fatigue, nutritional status, and CIN and vomiting-specific prognostic factors. Over three consecutive chemotherapy cycles, nausea was more prevalent than vomiting (47% vs. 12%). In chemotherapy Cycle 1, intervention participants reported significantly better QoL related to CIN (*p* = 0.029), chemotherapy-induced nausea and vomiting (CINV)-related QoL (*p* = 0.043), global QoL (*p* = 0.015) and less fatigue (*p* = 0.006) than placebo participants. There were no significant results in Cycle 2. In Cycle 3, global QoL (*p* = 0.040) and fatigue (*p* = 0.013) were significantly better in the intervention group compared to placebo. This trial suggests adjuvant ginger supplementation is associated with better chemotherapy-induced nausea-related quality of life and less cancer-related fatigue, with no difference in adverse effects compared to placebo.

## 1. Introduction

The prevention and management of chemotherapy-induced nausea and vomiting (CINV) is a priority in the oncology setting. While the development of a range of anti-emetic medications has reduced the prevalence of CINV, vomiting and, in particular, nausea, are still experienced by up to 25% and 61% of cancer patients, respectively [1]. CINV is also associated with poor quality of life (QoL) and malnutrition and if persistent, CINV can result in cancer therapy delays and dose reductions, culminating in poorer treatment outcomes [2,3,4]. Furthermore, when nausea and vomiting are measured separately, nausea is reported to affect QoL to a greater extent than vomiting. This suggests that additional interventions to control nausea are required [3].

Various interventions to reduce CINV have been investigated. These include pharmaceuticals (e.g., olanzapine), behavioral interventions (e.g., progressive muscle relaxation), and nutraceuticals such as ginger supplementation [5,6,7]. The compounds within ginger are understood to possess multiple properties relevant to the management of CINV. These include 5-Hydroxytryptamine_3_ receptor antagonism, which is also one of the cornerstones of modern antiemetic drug therapies such as ondansetron and granisetron [8].

Ginger has been trialed with some success for other types of nausea, including morning sickness and post-operative nausea and vomiting [9,10,11]. There is also mounting evidence supporting the use of adjuvant ginger to reduce CINV [7]. However, as discussed in our previous articles [7,12], extant research has multiple methodological limitations that must be addressed before this intervention can be recommended as a complement to routine clinical practice. These limitations include the lack of control for prognostic factors, potentially suboptimal dosing regimens, and inconsistent use of validated questionnaires and standardized ginger products [7,12]. The current study was designed to overcome these limitations.

The primary aim of this double-blind, randomized placebo-controlled trial was to determine the effect of adjuvant time- and dose-standardized ginger on chemotherapy-induced nausea (CIN)-related QoL, compared to placebo, in chemotherapy-naïve patients over three cycles of moderately- or highly-emetogenic chemotherapy.

Previously unexplored, but clinically important outcomes, were also investigated. These include cancer-related fatigue and malnutrition; both prevalent in chemotherapy cohorts, and consistently associated with CINV, and linked with significant decrements in patient QoL [2,13]. In addition, the potential correlation between ginger and the anti-emetic medication aprepitant was assessed. This was prompted by previous research that reported worse control of delayed CINV in patients receiving 2 g of ginger and aprepitant [14].

## 2. Methods

The design of this double-blind, randomised placebo-controlled trial is based on our fully detailed protocol manuscript [12]. The primary outcome and sample size calculation, however, has been amended since publication of our protocol manuscript. The study protocol was approved by the Metro South Human Research Ethics Committee, Brisbane, Australia and the Bond University Human Research Ethics Committee, Gold Coast, Australia. This trial was also registered with the Australian New Zealand Clinical Trials Registry (ACTRN12613000120774).

### 2.1. Sample and Recruitment

Patients were recruited if they were chemotherapy-naïve, were due to receive a moderately- or highly-emetogenic chemotherapy regimen, were at least 18 years old, had a baseline Karnofsky score >60 [15], had no known concurrent neoplasms or illnesses that induced nausea independent of chemotherapy, and did not self-prescribe therapies or complementary products used for nausea. Patients were excluded if they were scheduled to receive radiotherapy during the study period, were pregnant or lactating, concurrently used other ginger-containing supplements or ingested large quantities of ginger, had a history of adverse reactions to ginger, and/or thrombocytopenia. These inclusion and exclusion criteria were applied equally to both the intervention and placebo groups. Chemotherapy regimens were categorized as highly- or moderately-emetogenic consistent with the Multinational Association for Supportive Care and Cancer anti-emetic guidelines [16]. Written informed consent was obtained at time of enrolment.

Patients were recruited from the Princess Alexandra Hospital, Brisbane, Australia from March 2014 to February 2015. Potentially eligible patients were identified by research staff during daily chemotherapy education sessions and through the hospital chemotherapy scheduling system within one week prior to the first cycle of chemotherapy.

### 2.2. Intervention

Participants were randomly assigned to receive either 1.2 g (4 × 300 mg) of a standardized ginger extract or placebo in conjunction with the standard antiemetic therapy prescribed by their physician. The rationale for the selected dose is based on the dose used in previous trials that have reported significant improvements, pharmacokinetic data, and concerns regarding possible contraindications at high doses, as described in our previous protocol manuscript [12]. The ginger extract was standardized to contain 5% gingerols in capsule form. Each capsule, containing 300 mg of ginger extract with 15 mg of active ingredient per capsule (60 mg per 1.2 g), was double encapsulated to enhance patient blinding. Placebos were prepared with an inert filler and capsules that matched the intervention. The gingerol and shogaol content of the ginger extract was independently analyzed at the beginning and end of the trial by Southern Cross Plant Science Department at Southern Cross University and Bond University, respectively, using high performance liquid chromatography to ensure consistent potency of the intervention.

### 2.3. Procedure

Eligible patients were randomly allocated to ginger or placebo capsules by an independent company using a computer-generated sequence. Participants received three questionnaire booklets, one for each cycle of chemotherapy, which were either mailed back to the researchers upon completion or collected during the following cycle. All staff members involved in recruitment and outcome assessment were blinded to the results of randomization. Participants were followed over three chemotherapy cycles in order to evaluate the effect of the intervention over an extended period of their chemotherapy treatment. For each cycle, outcomes were assessed 3 days prior to chemotherapy until 4 days post-chemotherapy (i.e., over 7 days). Participants were asked to consume the study capsules 4 times per day, with each meal, for 5 days per chemotherapy cycle, commencing on the day of chemotherapy.

### 2.4. Outcome Measures

#### 2.4.1. Primary Outcome

The primary outcome was chemotherapy-induced nausea-related (CIN) quality of life (QoL) based on the rationale that this outcome is more clinically meaningful compared to other standard measures of CINV that may not fully reflect the impact of reported CINV on all domains of daily living. For example, a patient may report a low frequency of nausea but this may significantly impact on their QoL. This was measured using the Functional Living Index Emesis 5 Day Recall (FLIE-5DR) questionnaire, a validated measure of the impact of CINV on patients’ general well-being [17]. It comprises eighteen questions on 7-point Likert scales that assess the separate effects of nausea and vomiting on QoL. Scores can range from 9 to 63 for each domain (i.e., nausea or vomiting) and 18 to 126 for the total CINV score. A higher score indicates better QoL. To the authors’ knowledge, no minimal clinically important difference has been established for the FLIE-5DR; however, using the parameters established by Martin et al. [18], “no impact on daily life” was defined as an average item score greater than 6 on the 7-point scale. Therefore, a total score greater than 108 (out of a total score of 126) and a domain-specific score of 54 (out of a total score of 63) meant that CINV had minimal impact on daily life. Participants completed this questionnaire twice per chemotherapy cycle, at baseline and 4-days post-chemotherapy.

#### 2.4.2. Secondary Outcomes

A total score for CINV as well as separate scores for nausea, retching and vomiting were elicited using the validated, 8-item self-report tool, the Rhodes Inventory of Nausea, Vomiting and Retching (INVR) [19]. The INVR assesses the frequency, duration and severity of nausea, vomiting and retching. It provides domain-specific scores for nausea, vomiting and retching as well as a total score for CINV calculated from the combined domains.

The following operational definitions defined each phase of CINV. Anticipatory CINV was defined as any symptom occurring in the 24 h prior to chemotherapy administration [20]. Acute CINV was defined as any nausea and/or vomiting symptoms that occurred within 24 h of the administration of chemotherapy, while delayed CINV was defined as any nausea and/or vomiting symptoms that occurred after the acute phase and for the following 5 days [20]. In order to measure each of these phases of CINV, the INVR was administered one day before the commencement of chemotherapy (anticipatory CINV), on the day of chemotherapy (acute CINV) and during each of the 4 proceeding days to assess delayed CINV.

The INVR is designed to measure symptoms over a 12-h period; however, to minimize survey burden, this period was extended to 24 h so that participants would only need to complete one questionnaire per 24 h. For each 24-h period, a score (between 3 to 15 for nausea and vomiting, 2 to 10 for retching) is given for each symptom and a total score is derived from each symptom score (from 8 to 40). For delayed symptoms, scores from the three 24 hour periods after the acute phase were combined. 

Nutritional status was assessed once per chemotherapy cycle, on the day of chemotherapy, by an appropriately-trained research dietitian using the Patient-generated Subjective Global Assessment (PG-SGA) tool [21]. The PG-SGA provides a global rating of either A (well nourished), B (suspected or moderately malnourished) or C (severely malnourished), as well as a continuous score that increases with the severity of symptoms and the concomitant need for symptom management.

Global cancer-related QoL and cancer-related fatigue were assessed at baseline and 4-days post-chemotherapy using the Functional Assessment of Cancer Therapy-Global (FACT-G) and the Functional Assessment of Chronic Illness Therapy-Fatigue (FACIT-F) assessment questionnaires, respectively [22,23]. Both questionnaires are valid and widely-used within the cancer setting. The FACT-G is a self-report questionnaire that contains 27 five-point Likert scales that assess four domains of global (as opposed to CINV-specific) QoL. These are physical well-being, social/family well-being, emotional wellbeing, and functional well-being. The FACIT-F comprises 13 five-point Likert scales, and was used to capture self-reported symptoms of fatigue before and after each chemotherapy cycle. Possible scores for the FACT-G and FACIT-F range from 0 to 108 and 0 to 52, respectively, with higher scores indicating better QoL and less fatigue. A four-point difference between groups in FACT-G scores was considered a clinically meaningful difference [24]. Participants were deemed clinically fatigued if they reported a FACIT-F score ≤34, with a difference of 3 points between groups considered a clinically meaningful difference [25,26].

A new questionnaire was developed as part of this project to assess the prevalence of prognostic factors identified to increase the risk of CINV. The rationale for implementing this questionnaire was that an uneven distribution of these prognostic factors between the intervention and placebo groups could influence the results. The questionnaire included five items that assessed the patient’s history of morning sickness and motion sickness, their average weekly alcohol intake and their history of anxiety [27,28]. Additional prognostic factors including age, gender and the emetogenicity of the chemotherapy regimen were retrieved during the initial patient interview.

To determine adherence to the study protocol, participants were asked to record the number of capsules consumed each day during the study period. The quality of patient blinding was assessed at the end of each chemotherapy cycle during participant interviews, in which participants were asked to state the capsule (placebo or ginger) they believed they had received.

### 2.5. Adverse Events

Safety concerns and adverse events were monitored via telephone interviews during each cycle, 5-day post-chemotherapy. Participants were asked about any hospitalizations or adverse events during the study period. To assess any negative effects of ginger supplementation, between-group differences in a range of symptoms were also assessed using the Edmonton Symptom Assessment Scale, which was administered at each cycle of chemotherapy, at baseline and 5-day post-chemotherapy [29]. This is a validated 10-item questionnaire that measures the severity of common symptoms experienced by cancer patients including pain, anxiety and drowsiness.

### 2.6. Statistical Analysis

Statistical analysis was conducted using SPSS Version 20^®^ (SPSS Inc., Chicago, IL, USA). Descriptive analysis of baseline participant characteristics was undertaken. Bivariate outcomes were assessed using Chi-square analysis. Normally distributed continuous outcomes were assessed using independent sample t-tests. The Mann-Whitney U test was used for non-parametric outcomes. Data were analyzed on an intention-to-treat basis. A *p* value < 0.05 indicated statistical significance. Mean and standard deviation (SD) were reported for normally distributed data. Median with 25th percentile and 75th percentile were reported for non-parametric data. Missing data were handled using multiple imputation. In order to explore the association between ginger supplementation combined with aprepitant and worse delayed-CINV, a subgroup analysis in patients receiving aprepitant was also conducted.

Sample size was calculated based on the ability to detect a clinically meaningful difference in the primary outcome of nausea-related QoL. Hence, using the standard deviations from a preliminary feasibility study of ten participants and a desired mean difference of 9 points on the nausea-related subdomain of the FLIE-5DR, a sample size of 82 was estimated to provide sufficient power to detect a statistically and clinically significant difference in nausea-related QoL with 80% power and 5% significance.

## 3. Results

### 3.1. Patient Demographics and Adherence

**Figure 1:**
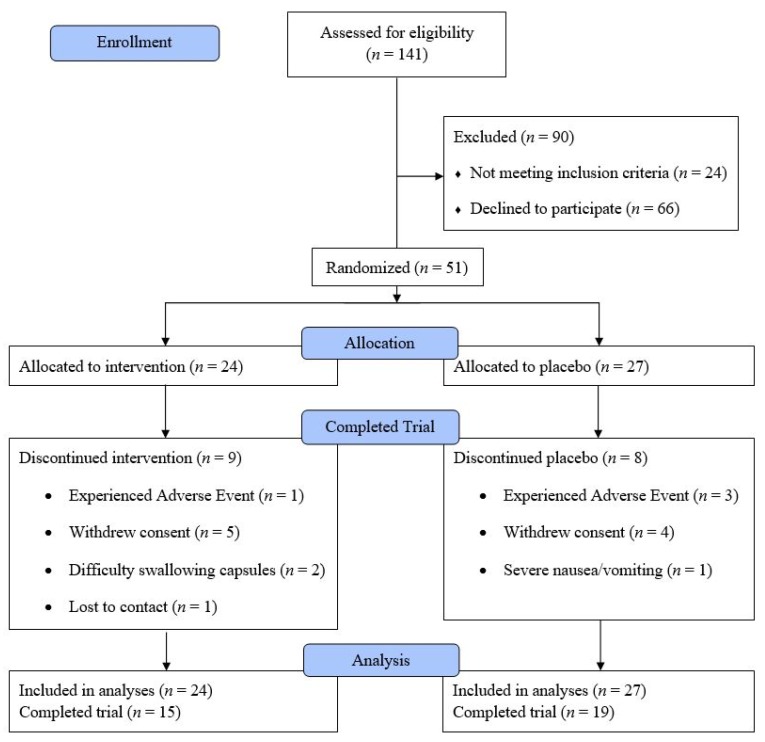
CONSORT (Consolidated Standards of Reporting Trials) flow diagram.

Fifty-one patients were enrolled in this study, of which 34 completed all three cycles (Figure 1). There were no significant differences in baseline patient characteristics between the intervention and placebo group (*p* > 0.05 for all characteristics). The majority of patients (84%) were scheduled to undergo moderately emetogenic chemotherapy regimens (Table 1).

### 3.2. CINV-Related Quality of Life

After cycle 1, participants assigned to the intervention group reported better nausea-related QoL (Median (25th, 75th percentile) = 61.5 (56.2, 63) vs. 54 (46, 63); *p* = 0.029) and better total CINV-related QoL (Median (25th, 75th percentile) = 124.5 (113.2, 126) vs. 111 (99, 126); *p* = 0.043) compared to patients assigned the placebo. Examination of median CINV- and nausea-related QoL at Cycle 1 in the placebo and intervention groups suggests that the clinical significance of this effect was minimal in both groups (Table 2). No other significant effect was detected for vomiting-related QoL or for any outcome at cycles 2 and 3.

### 3.3. Nausea and Vomiting Symptoms

Over the three chemotherapy cycles, acute and delayed CINV occurred in 39% and 65% of all participants (Table 3). In both groups, nausea was more common than vomiting during each cycle, with 47% vs. 12% of participants overall reporting symptoms during at least one cycle, respectively. There were no significant differences in the prevalence and score of CINV between the intervention and placebo group at any time point.

In a subgroup analysis of participants (*n* = 18) assigned to the intervention with and without being prescribed aprepitant, there were no statistically significant differences in CINV between groups at any time point.

### 3.4. Fatigue, Nutrition Status, and Cancer-Related Quality of Life

Clinically significant fatigue and malnutrition were experienced by 35% and 22% of participants over the study period. Ginger supplementation was associated with improved measures of chemotherapy-related fatigue in Cycle 1 (Mean ± SD = 41.8 ± 13 vs. 32.2 ± 10.8; *p* = 0.006) and Cycle 3 (Mean ± SD = 42.4 ± 10.2 vs. 36.1 ± 7.2; *p* = 0.013) compared to placebo. There was also a statistically significant difference in cancer-related QoL at Cycle 1 (Mean ± SD = 85.1 ± 18.9 vs. 71.9 ± 18.3; *p* = 0.015) and Cycle 3 (Mean ± SD = 83.6 ± 15.0 vs. 75.1 ± 13.9; *p* = 0.040).

Each of the significant associations reported for the cancer-related QoL (>4 point difference) and cancer-related fatigue (>3 point difference) were clinically important. No significant difference in nutritional status was detected between the intervention and placebo group during the study period (*p* > 0.05; Table 2).

### 3.5. Participant Blinding and Adherence

More participants in the intervention group were able to correctly guess their assigned group when compared to participants in the placebo group (63% compared to 30%, respectively). For participants in the intervention group who successfully identified their allocation, the most common rationale provided was a lack of nausea (60%), the smell of the capsules (20%), and ginger taste or reflux (13%). Adherence to the study intervention was moderate-to-high, with 71% of all participants (67% in ginger group and 74% in placebo group) consuming at least 3 of the 4 prescribed capsules per day.

### 3.6. Effect of Prognostic Factors on CINV-Related Outcomes

The hypothesized prognostic factors of age, gender, anticipatory CINV, and chemotherapy emetogenicity were analyzed with no significant associations detected (*p* > 0.05) between these variables and any measure of CINV.

### 3.7. Adverse Events

Four patients in this trial experienced significant adverse events, none of which could reasonably be attributed to the ginger intervention. These include one participant whose lung collapsed, one allergic reaction to pegfilgrastim, and two emergency room admissions due to neutropenic fever. Three of the four adverse events occurred within the placebo group. The most commonly reported side-effects in the intervention group included constipation and reflux, which were reported by two and four participants, respectively.

### 3.8. Incomplete Questionnaires

Few participants completed the Edmonton Symptom Assessment Scale and CINV-prognostic questionnaire; hence, the results from these questionnaires were not statistically meaningful.

## 4. Discussion

The results of this study indicate that, compared to placebo, adjuvant ginger is associated with a better nausea-related QoL, less cancer-related fatigue and better overall cancer-related QoL. Previous studies have reported that ginger reduces CINV; however, this is the first study to investigate whether this reduction translates into an improvement in QoL. As CINV has been demonstrated to significantly reduce QoL [17], this study provides evidence that ginger supplementation could be a viable adjuvant to traditional pharmacotherapy for CINV that enhances patients’ wellbeing during their cancer treatment. 

Despite the significant association of ginger and improved QoL, the findings indicate that there was no significant effect of ginger on the prevalence or severity of CINV. While the majority of previous research has reported that ginger supplementation reduces the incidence and severity of CINV, not all studies have reported benefits [14,31]. The prevalence of CINV during this study was high (39% and 65% of patients experienced acute and delayed CINV, respectively) which is consistent with the prevalence reported in other studies [1]. However, similar to the results of Fahimi et al., while the prevalence was high, the average score derived from the INVR was low in both the intervention and placebo groups [31]. This indicates that although a large proportion of participants experienced CINV, the average severity caused by these symptoms was low.

The statistically significant improvement in nausea-related QoL could also be clinically relevant. However, as there is no established minimal clinically important difference for the FLIE-5DR, the clinical significance of the better CINV- and nausea-related QoL in Cycle 1 reported in the intervention group is not easily elucidated. While the placebo group experienced poorer CINV- and nausea-related QoL, due to the generally high level of CINV- and nausea-related QoL in both intervention and placebo groups, it is difficult to determine the clinical significance of the ginger supplementation used in this trial with respect to these outcomes. Similarly, high ratings of QoL have been reported in previous observational studies [3,32]. A possible explanation for this is that this trial was conducted at a hospital that adheres to international anti-emetic guidelines and prescribes current generation anti-emetic medications such as aprepitant and granisetron. In contrast, many previous studies that have reported severe CINV were conducted before the wide-spread clinical use of these anti-emetics [33,34]. Furthermore, the process of “response shift” could have also influenced these results [35]. Response shift refers to the individual’s re-evaluation of the internal standards and the values with which an individual assesses their QoL, a process associated with repeat experiences of their treatment and its symptoms or comparison with other people’s experiences of it, which can appear comparatively worse than their own [35]. CINV-related QoL within the placebo group gradually improved (Table 2) which suggests that a “response shift” in participant’s assessment of QoL could have occurred over the course of their chemotherapy treatment.

The significant improvement in fatigue reported in this study corroborates the results of previous clinical trials where fatigue was reported at a significantly higher rate in the placebo group compared to the intervention group [14,36]. While the exact mechanism underpinning this finding is unknown, these results are hypothesis generating. Future mechanistic and clinical studies are needed to investigate the role of ginger in cancer-related fatigue.

Ginger supplementation was well-tolerated with no significant increase in adverse events and few side-effects reported. This is consistent with previous studies, which have reported minor adverse events [7]. Ginger has been reported in some (but not all) clinical studies to interfere with platelet aggregation [37]. During chemotherapy, this can potentially pose a significant concern due to the pre-existing risk of thrombocytopenia. Although there has been no indication of adverse clotting in this trial or previous studies, platelet function should be routinely monitored in this patient group [37].

Another potential concern is that ginger might reduce the effectiveness of anti-emetic therapy when patients are prescribed aprepitant. This was identified in a subgroup analysis in one study, which reported that patients who received 2 g of ginger and aprepitant experienced worse delayed CINV than patients who received 2 g of ginger without aprepitant [14]. This association, however, was not identified in patients prescribed aprepitant and a lower dose of ginger (1 g) indicating that this might only occur with higher doses (2 g) of ginger [14]. In the present study, participants receiving ginger supplementation and aprepitant reported neither statistically nor clinically significant differences in CINV. This relationship should continue to be investigated in larger trials to ensure that patients’ anti-emetic control is not compromised by ginger supplementation.

Despite our ginger supplements being doubly encapsulated, the most commonly reported side-effect in the intervention group was ginger taste or reflux. While this was considered a mild side-effect by participants, it is a potential confounding variable for clinical trials investigating ginger as the unique taste is likely to reduce the efficacy of blinding, which could influence subjective measures of nausea. In order to improve blinding efficacy, we recommended testing of blinding efficacy in a small sample before the commencement of recruitment.

This study has overcome limitations identified in previous studies by including the use of standardized extracts and chemical analysis of supplements that ensured potency throughout the study period, as well as the assessment of previously identified prognostic factors such as age and gender. Previous studies have not assessed the influence of these prognostic factors, which might have resulted in an imbalanced risk of CINV between the two treatment arms in these studies. Furthermore, in this study we controlled for anticipatory CINV, a conditioned response that develops via a pathway different to other types of CINV [28]. This was achieved by recognizing that the prevalence of anticipatory CINV increases with subsequent cycles; hence, only chemotherapy-naïve patients were recruited [38]. CINV was also assessed the day before each cycle of chemotherapy to capture anticipatory CINV. None of these prognostic factors were found to be associated with CINV-related outcomes, which could be linked to the small sample size.

A further strength of this study was the four-per-day dosing regimen, a process that accounted for the relatively short half-life of major ginger compounds, in contrast to the once or twice per day regimens adhered to in previous studies [39,40]. Based on the pharmacokinetics of ginger, we hypothesized that more frequent consumption of ginger would ensure sufficient plasma levels of the active compounds which could result in a greater level of effect [39]. Future studies that include additional arms are needed to determine the effect of different dosing regimens on the treatment effect.

We acknowledge the following limitations. First, there was a high level of attrition by Cycle 3 (33%) compared to some previous studies, which have reported an attrition rate of approximately 20% [14,31]. Due to the extended study time frame (3 cycles compared to 1,2), the increased number of capsules ingested required per day, and the expanded number of outcomes that were measured, the relatively high attrition could be because the trial protocol was overly burdensome to participants. This is also demonstrated by the low completion rates of some questionnaires (CINV prognostic and the Edmonton Symptom Assessment Scale questionnaires) which prevented meaningful statistical analysis of these outcomes. In future studies, it is recommended that particular consideration is taken to reduce the burden that is placed on participants by the study protocol such as by reducing the number of self-reported outcome measures.

Another limitation was that, while this trial was sufficiently powered to detect a significant difference in the primary outcome at Cycle 1, due to timing constraints and attrition, the trial did not meet the required sample size for the second and third cycles of chemotherapy. Introducing sufficient inflation factors in sample size calculations, as well as reducing study burden, is recommended in future studies to ensure sufficient power during subsequent cycles.

## 5. Conclusions

In summary, the results of this clinical trial suggest that compared to placebo, adjuvant ginger is associated with better chemotherapy-induced nausea-related and cancer-related quality of life, and less cancer-related fatigue. The results confirm several previous studies that report ginger supplementation to be well-tolerated and without significant side-effects. Further studies with larger sample sizes are required to confirm these results and to further explore the safety profile of ginger supplementation during chemotherapy.

## Figures and Tables

**Table 1:** Patient demographics at baseline.

	Total	Intervention Group	Control Group
*n*	51	24	27
Age (mean ± SD, years)	58 ± 12	57 ± 14	59 ± 11
Gender (*n*, % female)	32 (63)	16 (66)	16 (59)
Race (*n*, % Caucasian)	42 (82)	18 (75)	24 (88)
*Primary diagnoses (n)*			
Breast	13	7	6
Colon	19	8	11
Lymphoma	11	5	6
Other	8	4	4
*Chemotherapy Emetogenicity* (*n*)			
HEC	8.0	4.0	4.0
MEC	43.0	20.0	23.0
*Receiving aprepitant* (*n*)	18.0	7.0	11.0

HEC, Highly Emetogenic Chemotherapy. MEC, Moderately Emetogenic Chemotherapy. SD, standard deviation. These regimens were classified based on the Multinational Association of Supportive Care in Cancer guidelines [30].

**Table 2:** Cancer- and chemotherapy-induced nausea and vomiting (CINV)-related quality of life (QoL), cancer-related fatigue, and nutrition status.

	Cycle 1				Cycle 2				Cycle 3			
	Total	Placebo	Intervention	*p* Value	Total	Placebo	Intervention	*p* Value	Total	Placebo	Intervention	*p* Value
CINV-QoL	123 (103, 126)	111 (99, 126)	124.5 (113.2, 126)	0.043 *	124 (108, 126)	117 (109, 126)	124 (108, 126)	0.916	122 (107, 126)	120 (111, 126)	123.5 (107, 126)	0.931
Nausea-related QoL	60 (51, 63)	54 (46, 63)	61.5 (56.2, 63)	0.029 *	61 (49, 63)	56 (49, 63)	61 (52.2, 63)	0.494	56 (49, 63)	56 (49, 63)	56.5 (46.7, 63)	0.931
Vomiting-related QoL	63 (51, 63)	63 (51, 63)	63 (54, 63)	0.237	63 (51.9, 63)	63 (52, 63)	63 (54.7, 63)	0.663	63 (51, 63)	63 (54, 63)	59 (50.2, 63)	0.414
Global cancer-related QoL	78.1 ± 19.5	71.9 ± 18.3	85.1 ± 18.9	0.015 *	71.1 ± 14.5	67.6 ± 10.2	74.9 ± 17.7	0.077	79.1 ± 14.9	75 ± 13.8	83.6 ± 15	0.040 *
Fatigue	36.7 ± 12.8	32.2 ± 10.8	41.8 ± 13	0.006 *	36.1 ± 9.4	34.5 ± 7.9	37.7 ± 10.8	0.23	39.1 ± 9.2	36.1 ± 7.2	42.4 ± 10.2	0.013 *
Nutrition status at start of cycle (*n*, well nourished)	44	22	22	0.371	38	19	19	0.500	37	19	18	0.622

Normally distributed measures were presented as mean ± standard deviation and non-normally distributed measures were presented as median (25th percentile, 75th percentile). * signifies a statistically significant result (*p* < 0.05)

**Table 3:** Participant Inventory of Nausea, Vomiting and Retching (INVR) questionnaire scores and CINV prevalence.

	Cycle 1				Cycle 2				Cycle 3			
	Total	Placebo	Intervention	*p* Value	Total	Placebo	Intervention	*p* Value	Total	Placebo	Intervention	*p* Value
Anticipatory CINV score	8 (8, 9)	8 (8, 8)	8 (8, 8)	0.44	8 (8, 9)	8 (8, 9)	8 (8, 9)	0.61	8 (8, 8)	8 (8, 9)	8 ( 8,8)	0.76
Acute CINV score	9 (9, 11)	9 (9, 11)	10 (9, 10.7)	0.84	9 (9, 10)	9 (8, 10)	9 (9, 9)	0.94	9 (9, 11)	9 (9, 11)	10 (9, 11)	0.12
Vomiting score	3 (3, 3)	3 (3, 3)	3 (3, 3)	0.41	3 (3, 3)	3 (3, 3)	3 (3, 3)	0.99	3 (3, 3)	3 (3, 3)	3 (3, 3)	0.17
Nausea score	3 (3, 5)	3 (3, 5)	3.6 (3, 5)	0.46	3 (3, 4)	3 (3, 4)	3 (3, 3)	0.63	3 (3, 5)	3 (3, 5)	3 (3, 5)	0.79
Retching score	2 (2, 2)	2 (2, 2)	2 (2, 2)	0.78	2 (2, 2)	2 (2, 2)	2 (2, 2)	0.99	2 (2, 2)	2 (2, 2)	2 (2, 2)	0.46
Delayed CINV score	32 (27, 34)	32 (28, 34)	31 (26, 34.7)	0.74	30 (28, 31)	30 (29, 31)	29 (28, 31)	0.26	29 (27, 30)	29 (27, 30)	29 (27, 30)	0.86
Vomiting score	9 (9, 11)	9 (9, 12)	9 (9, 9.7)	0.74	9 (9, 10)	9 (9, 10)	9 (9, 10)	0.95	9 (9, 9)	9 (9, 10)	9 (9, 9)	0.69
Nausea score	13 (9, 19)	15 (9, 20)	11 (9, 17)	0.27	13 (9, 15)	12 (9, 16)	14.5 (9, 15)	0.54	12 (9, 16)	12 (9, 16)	12 (9, 16.5)	0.42
Retching score	6 (6, 6)	6 (6, 6)	6 (6, 6.05)	0.28	6 (6, 6)	6 (6, 6)	6 (6, 6.7)	0.56	6 (6, 6)	6 (6, 6)	6 (6, 6)	0.41

Normally distributed measures were presented as mean ± standard deviation and non-normally distributed measures were presented as median (25th percentile, 75th percentile).
